# The Simultaneous Efficient Recovery of Ammonia Nitrogen and Phosphate Resources in the Form of Struvite: Optimization and Potential Applications for the Electrochemical Reduction of NO_3_^−^

**DOI:** 10.3390/molecules29102185

**Published:** 2024-05-08

**Authors:** Liping Li, Jingtao Bi, Mengmeng Sun, Shizhao Wang, Xiaofu Guo, Fei Li, Jie Liu, Yingying Zhao

**Affiliations:** 1 Engineering Research Center of Seawater Utilization of Ministry of Education, School of Chemical Engineering and Technology, Hebei University of Technology, Tianjin 300401, China; liliping_z1027@163.com (L.L.); mengmeng_sun@hebut.edu.cn (M.S.); shizhaow@163.com (S.W.); 2009024@hebut.edu.cn (X.G.); lifei2008_ok@126.com (F.L.); liujie@hebut.edu.cn (J.L.); 2 Hebei Collaborative Innovation Center of Modern Marine Chemical Technology, Tianjin 300401, China; 3 Tianjin Key Laboratory of Chemical Process Safety, Tianjin 300130, China; 4 Shandong Technology Innovation Center of Seawater and Brine Efficient Utilization, Weifang 262737, China

**Keywords:** struvite, resource utilization, nitrogen recovery, phosphorus recovery, electrochemistry

## Abstract

In response to the need for improvement in the utilization of ammonium-rich solutions after the electrochemical reduction of nitrate (NO_3_^−^–RR), this study combined phosphorus-containing wastewater and adopted the electrochemical precipitation method for the preparation of struvite (MAP) to simultaneously recover nitrogen and phosphorus resources. At a current density of 5 mA·cm^−2^ and an initial solution pH of 7.0, the recovery efficiencies for nitrogen and phosphorus can reach 47.15% and 88.66%, respectively. Under various experimental conditions, the generated struvite (MgNH_4_PO_4_·6H_2_O) exhibits a typical long prismatic structure. In solutions containing nitrate and nitrite, the coexisting ions have no significant effect on the final product, struvite. Finally, the characterization of the precipitate product by X-ray diffraction (XRD) revealed that its main component is struvite, with a high purity reaching 93.24%. Overall, this system can effectively recover ammonium nitrogen from the NO_3_^−^–RR solution system after nitrate reduction, with certain application prospects for the recovery of ammonium nitrogen and phosphate.

## 1. Introduction

In recent years, the presence of large amounts of nitrate in surface water and groundwater has posed a significant threat to the ecological environment and human health [1,2,3]. Removing nitrates from environments such as surface water and groundwater is crucial for preventing such problems. Among them, electrochemical methods have emerged as a promising technique for nitrate treatment due to their simplicity, high efficiency, and lack of secondary pollution [4,5,6].

The main products of the electrochemical reduction of nitrate are mainly nitrogen gas (N_2_) or ammonium nitrogen (NH_4_^+^–N) [7,8]. Nitrogen gas is formed and directly released into the air in a non-toxic and harmless form, while the utilization form and pathway of ammonium nitrogen existing in the solution need to be improved. Ammonia stripping, ion exchange, membrane separation, crystallization precipitation, and other technologies are commonly used for ammonia nitrogen recovery [9,10]. Among them, the crystallization precipitation method of struvite has become a promising method for recovering ammonia nitrogen due to its advantages of simple operation, high efficiency, and effective reduction in treatment costs [11,12]. For instance, Huang demonstrated the feasibility of simultaneously recovering phosphate and removing ammonia nitrogen from piggery wastewater using a coupled electrochemical process. Under optimal experimental conditions, the removal rate of ammonia nitrogen in wastewater can exceed 90% [13]. Kruk reported that struvite precipitation using a magnesium sacrificial anode as the source of magnesium allowed actual phosphorus removal and direct recovery as struvite [14]. The electrochemical precipitation method for preparing struvite not only enables the effective recovery of ammonia nitrogen but also removes phosphate from wastewater.

Therefore, combining the ammonia nitrogen solution after NO_3_^−^–RR with phosphorus-containing wastewater to construct an electrochemical struvite crystallization precipitation process system not only efficiently utilizes low-grade ammonia nitrogen but also achieves phosphorus removal, thereby achieving the synergistic and efficient treatment of ammonia nitrogen and phosphate. The resulting struvite precipitate can also serve as a slow-release agricultural fertilizer [15,16], enhancing the economic viability of the recovery process system.

This study intended to utilize this electrochemical method to investigate the effects of factors such as the current density, initial pH, and coexisting ions on the recovery of the ammonia nitrogen solution after NO_3_^−^–RR and the formation of the struvite precipitate. Key parameters affecting the process flow were evaluated from the aspects of nitrogen and phosphorus recovery rates and the purity of the struvite precipitate, and XRD and SEM were used for the characterization and analysis of the precipitated crystals. The successful application of the recovery process system achieved the efficient removal of phosphate in phosphorus-containing wastewater and the high-efficiency recovery and utilization of ammonia nitrogen resources in the solution after nitrate reduction, providing new insights for the development of ammonia nitrogen resource recovery technology.

## 2. Results and Discussion

### 2.1. The Impact of the Current Density

**Figure 1:**
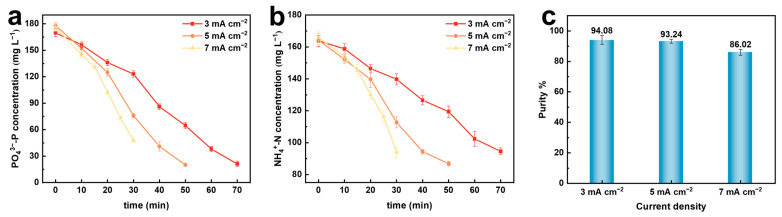
Effect of different current densities on electrochemical recovery of nitrogen and phosphorus. (**a**) PO_4_^3−^–P conversion rate; (**b**) NH_4_^+^–N conversion rate; (**c**) purity of struvite precipitate.

The current density determines the rate at which magnesium ions are dissolved from the magnesium electrode, directly affecting the ratio of magnesium ions to nitrogen and phosphorus concentrations in the solution, thereby influencing the efficiency of struvite precipitation in the solution [17,18]. Therefore, the influence of the current density on the recovery of nitrogen and phosphorus resources and the purity of the formed struvite was studied by the electrochemical precipitation method in 50 mL of a mixed solution of 160 mg·L^−1^ NH_4_^+^–N and 176 mg·L^−1^ PO_4_^3−^–P. The experimental conditions were as follows: initial pH: 7.0; initial PO_4_^3−^–P concentration: 176 mg·L^−1^; concentration of other coexisting ions: 0 mg·L^−1^. The experimental results are shown in Figure 1.

In Figure 1a,b, it can be observed that with the increase in the current density, the concentrations of PO_4_^3−^–P and NH_4_^+^–N decrease with time. This is because, at higher current densities, more magnesium ions are released from the magnesium anode within the same period, prompting the struvite precipitation reaction to move in the positive direction. Therefore, the rate of formation of struvite when combining ammonium nitrogen and phosphate in the solution is faster. Comparatively, at a higher current density, the slope of the ion concentration change curve is greater, indicating a faster recovery rate of phosphate and ammonium nitrogen. From the figure, it can be seen that as the current density increases from 3 mA·cm^−2^ to 7 mA·cm^−2^, the recovery rates of ammonium nitrogen and phosphate also increase accordingly. A purity analysis of the precipitates formed at different current densities (Figure 1c) shows that as the current density increases, the purity of the precipitate decreases from 94.08% to 86.02%. An excessive current density may have a negative impact on electrolysis efficiency. The hydrogen evolution reaction occurring at the cathode increases the pH near the cathode, and the oxidation reaction at the anode leads to corrosion, with the formation of a stable oxide layer on the anode surface causing passivation effects. This passivation effect increases resistance, ultimately leading to higher operating costs and reduced electrolysis efficiency [19,20]. Although the purity of the precipitates is highest at a current density of 3 mA·cm^−2^, achieving the same recovery rate requires a longer time, increasing the energy consumption of the reaction.

**Figure 2:**
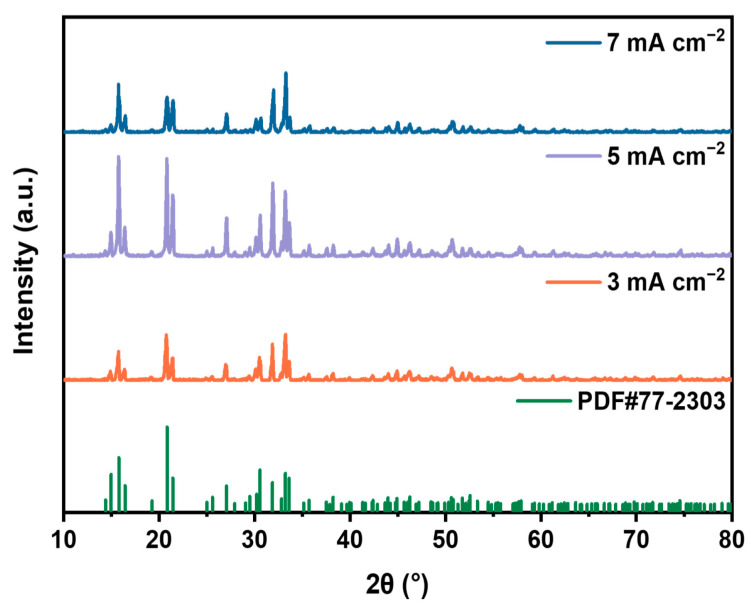
XRD patterns of precipitated products at different current densities.

Additionally, the characterization of the precipitates was conducted via XRD analysis (Figure 2), revealing that the peak positions and intensities of the XRD patterns of the precipitates closely match those of standard struvite, indicating the presence of struvite in all precipitates under various experimental conditions. This confirms the formation of struvite precipitates in the electrochemical system. Moreover, at a current density of 5 mA·cm^−2^, the peak intensity of struvite is higher and sharper, suggesting the highest degree of crystallinity at this current density.

Through a study at different current densities, it was found that at a current density of 5 mA·cm^−2^, the struvite purity is highest, accompanied by maximum electrolysis efficiency and minimal energy consumption. Therefore, a current density of 5 mA·cm^−2^ was selected for subsequent experiments.

### 2.2. The Impact of the Initial pH

**Figure 3:**
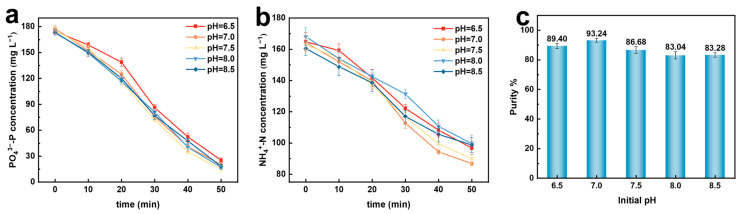
Effect of different initial pH on electrochemical recovery of nitrogen and phosphorus. (**a**) PO_4_^3−^–P conversion rate; (**b**) NH_4_^+^–N conversion rate; (**c**) purity of struvite precipitation.

The initial pH of the solution is an important indicator affecting the electrochemical precipitation method for the struvite recovery of nitrogen and phosphorus resources. Different forms of NH_4_^+^ and PO_4_^3−^ ions exist at different pH values, leading to differences in the types and morphologies of the precipitated products [21,22]. The influence of the initial pH on the electrochemical precipitation method for the struvite recovery of nitrogen and phosphorus resources was investigated, with an initial current density of 5 mA·cm^−2^, electrolysis time of 50 min, initial PO_4_^3−^–P of 176 mg·L^−1^, and other coexisting ion concentrations of 0 mg·L^−1^. The experimental results are shown in Figure 3.

With the variation in the initial solution pH, there is no significant change in the phosphate concentration in the solution, and at the end of the 50 min reaction, it decreases to similar values (Figure 3a). However, the decrease in the ammonium nitrogen concentration shows a trend of initially increasing and then decreasing with the increase in the initial pH, reaching the maximum recovery rate at pH = 7.0 (Figure 3b). This is mainly due to the relationship between the decrease in phosphate and ammonium nitrogen concentrations and the conditional solubility product of struvite. At higher pH values, Mg^2+^ reacts with OH^−^ in the solution to generate Mg(OH)_2_ precipitates, while NH_4_^+^ reacts with OH^−^ to generate NH_3_, thereby affecting the recovery efficiency of ammonium nitrogen and phosphate [23].

In Figure 3c, it can also be observed that the purity of struvite in the final precipitate shows a trend of initially increasing and then decreasing with the increase in the initial pH, which is consistent with the variation trend of the ammonium nitrogen concentration in the solution. At a lower pH of 6.5, phosphorus (P) mainly exists in the form of H_2_PO_4_^−^ and HPO_4_^2−^ in the solution [24,25], which does not reach the optimal conditions for producing struvite precipitates, resulting in a slower rate of struvite crystallization and possibly the precipitation of other phosphate by-products such as Mg(H_2_PO_4_)_2_. However, when the initial pH is above 7.0, the concentration of OH^−^ in the initial reaction solution increases, leading to a final solution pH greater than 10 after the reaction. At this point, P mainly exists in the form of PO_4_^3−^, and the pH exceeds the optimal pH for struvite formation [26]. This may also result in the appearance of other by-products, such as Mg(OH)_2_ and Mg_3_(PO_4_)_2_, and the ammonium nitrogen in the solution may combine with OH^−^ to form ammonia gas, leading to a decrease in the purity of the final struvite [23].

**Figure 4:**
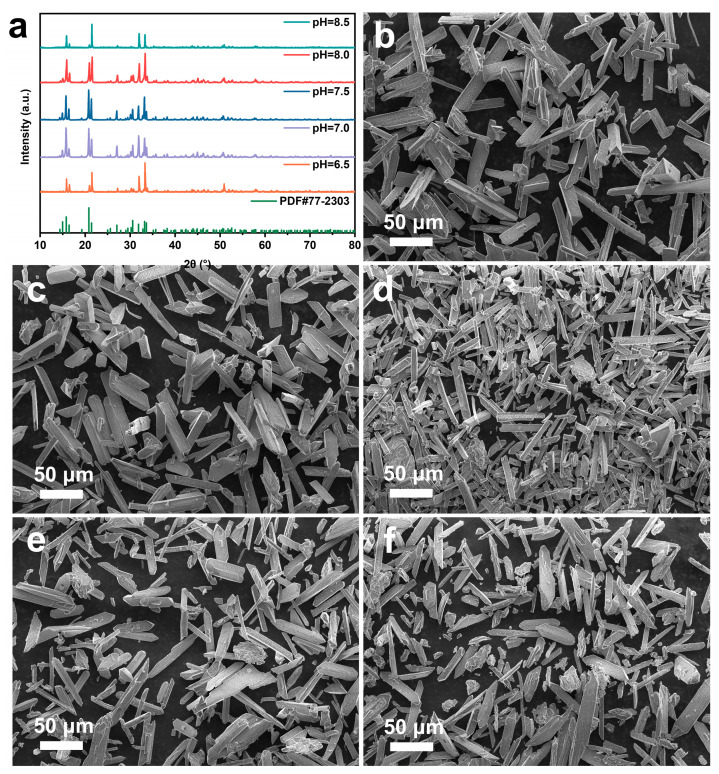
Precipitate products at different initial pH. (**a**) XRD patterns; SEM images of precipitates formed at pH (**b**) 6.5; (**c**) 7.0; (**d**) 7.5; (**e**) 8.0; (**f**) 8.5.

As shown in Figure 4a, the peak positions and heights of the XRD patterns of various precipitates are similar to those of standard struvite, indicating that struvite was formed in the precipitates under various experimental conditions, and the peak intensity gradually decreased with increasing pH. An SEM characterization analysis of the precipitates (Figure 4b–f) revealed that the precipitates exhibited typical elongated prismatic structures. With increasing pH, a small number of blocky crystals gradually appeared, consistent with literature reports [27].

At an initial solution pH of 7.0, the efficient and rapid recovery and conversion of phosphate and ammonium nitrogen resources can be achieved. Therefore, pH = 7.0 was chosen for subsequent experiments.

### 2.3. The Impact of Coexisting Ions in Nitrate Reduction Wastewater

In previous studies, the electrochemical reduction of nitrate has effectively treated nitrate-containing wastewater. The main reaction products are primarily present in the solution in the form of ammonium nitrogen (NH_4_^+^–N), but there are also small amounts of unreacted nitrate (NO_3_^−^–N) and nitrite (NO_2_^−^–N). Therefore, the influence of coexisting ions in the solution after the electrochemical reduction of nitrate on the electrochemical precipitation preparation of struvite for nitrogen and phosphorus recovery was investigated.

#### 2.3.1. The Effect of Nitrate

The influence of the nitrate concentration in the solution after the electrochemical reduction of nitrate is relatively small. Therefore, the effect of 0, 5, 10, 15, and 20 mg·L^−1^ NO_3_^−^–N on the preparation of struvite by the electrochemical precipitation method was investigated.

**Figure 5:**
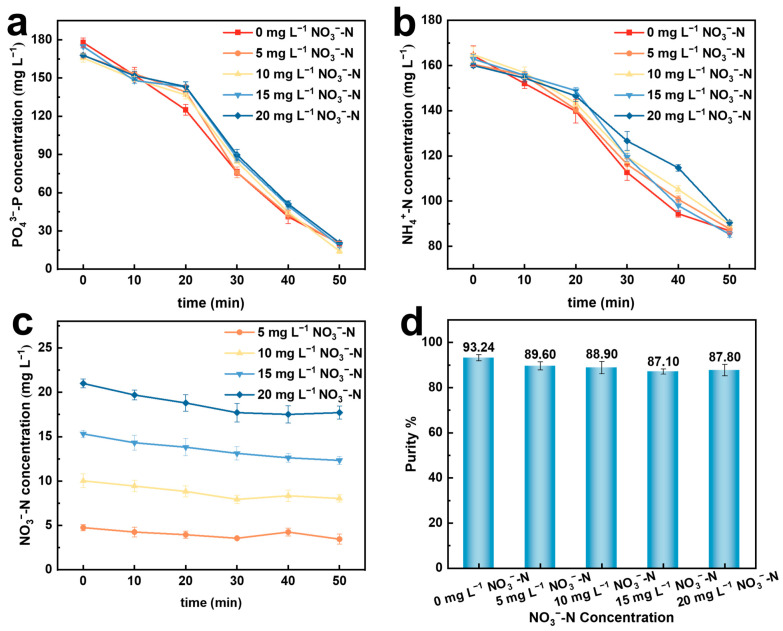
Effects of different initial NO_3_^−^–N concentrations on electrochemical recovery of nitrogen and phosphorus. (**a**) PO_4_^3−^–P conversion rate; (**b**) NH_4_^+^–N conversion rate; (**c**) changes in NO_3_^−^–N concentration; (**d**) purity of struvite precipitation.

**Figure 6:**
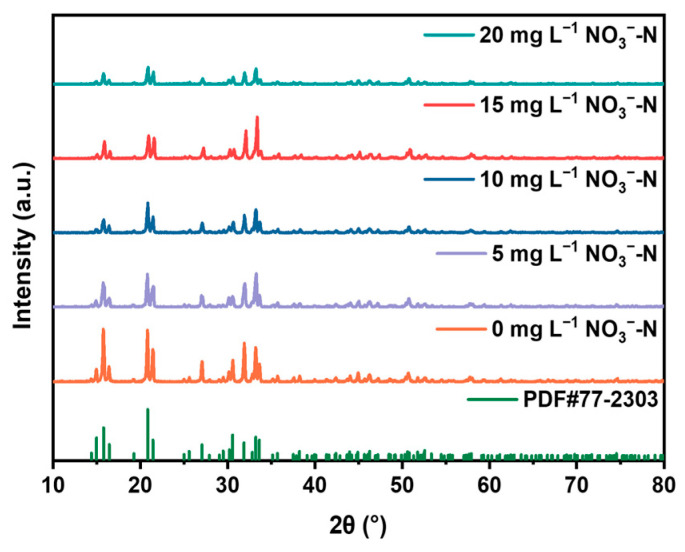
XRD patterns of precipitated products at different initial NO_3_^−^–N concentrations.

In Figure 5a,b, it can be observed that the addition of nitrate has little effect on the final recovery rate of phosphate, but as the concentration of nitrate increases, the recovery rate of phosphate decreases to some extent. This may be because the presence of nitrate during the formation of struvite crystals affects the binding of phosphate to ammonium nitrogen, and the slowed recovery rate leads to the occurrence of other side reactions during the formation of struvite crystals, resulting in phosphate precipitation. With the addition of nitrate, the recovery rate of ammonium nitrogen also remains largely unchanged, but it does have a noticeable impact on the recovery rate. The higher the concentration of nitrate, the more significant the slowdown in the recovery rate of ammonium nitrogen. This may be because nitrate, being negatively charged, hinders the binding of positively charged ammonium nitrogen to phosphate, thereby affecting the rate of formation of struvite crystal precipitates, resulting in a slight decrease in struvite purity (Figure 5d). Figure 5c shows the change in NO_3_^−^–N concentration during the electrochemical precipitation process. There is no significant change in the concentration of NO_3_^−^–N during the reaction process, indicating that NO_3_^−^–N does not participate in the electrochemical reaction. The XRD analysis in Figure 6 indicates that struvite is present in all precipitates.

#### 2.3.2. The Effect of Nitrite

In the solution, after the electrochemical reduction of nitrate, there are still some unreacted intermediate products: nitrites. Therefore, the influence of 0, 10, 15, 20, and 30 mg·L^−1^ NO_2_^−^–N on the preparation of struvite by the electrochemical precipitation method was investigated.

**Figure 7:**
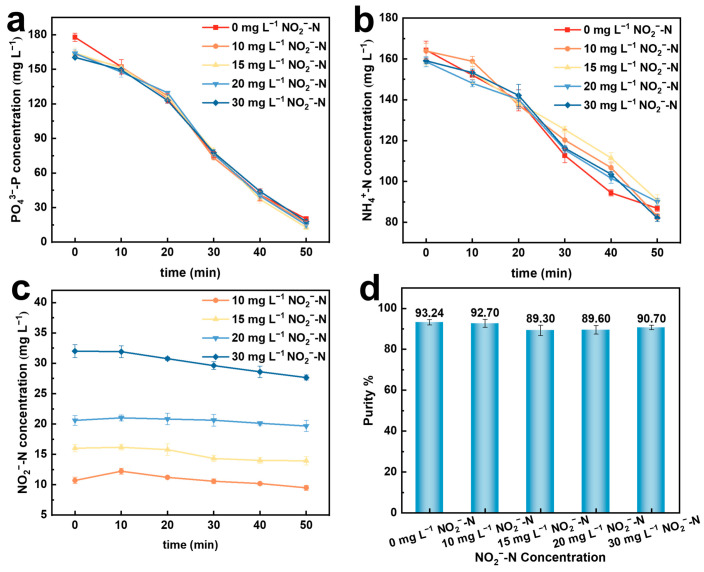
Effects of different initial NO_2_^−^–N concentrations on the electrochemical recovery of nitrogen and phosphorus. (**a**) PO_4_^3−^–P conversion rate; (**b**) NH_4_^+^–N conversion rate; (**c**) changes in NO_2_^−^–N concentration; (**d**) purity of struvite precipitation.

**Figure 8:**
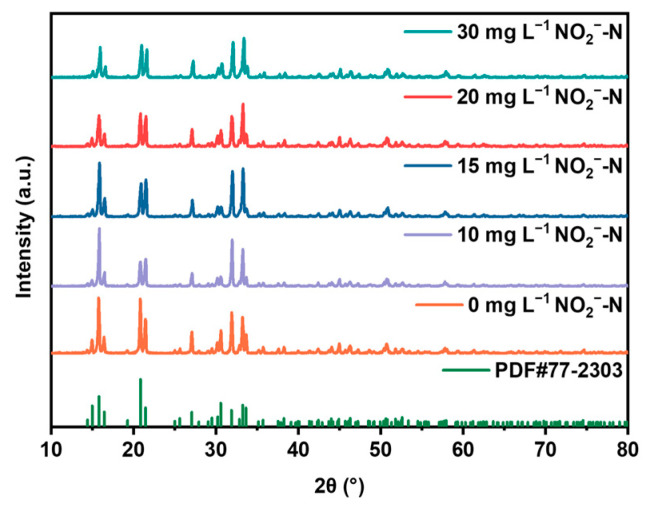
XRD patterns of precipitated products at different initial NO_2_^−^–N concentrations.

As shown in Figure 7a,b, with the increase in nitrite concentration, there is no significant impact on the recovery rate of phosphate. However, the presence of nitrite ions affects the recovery rate of ammonium nitrogen, but it has little effect on the final recovery rate of ammonium nitrogen. That is, the negatively charged NO_2_^−^–N has a certain inhibitory effect on the binding of phosphate ions to ammonium nitrogen, resulting in a slight decrease in the final struvite precipitate purity (Figure 7d). Figure 7c indicates that there is little change in the concentration of NO_2_^−^–N during the reaction process, indicating that NO_2_^−^–N does not participate in the electrochemical precipitation reaction. The XRD analysis in Figure 8 shows that struvite is present in all precipitates.

In the presence of coexisting NO_3_^−^ and NO_2_^−^ ions, the recovery rates for nitrogen and phosphorus were not significantly affected. However, the coexisting ions may slightly decrease the purity of the struvite precipitation product.

### 2.4. The Impact of Coexisting Metal Cations

**Figure 9:**
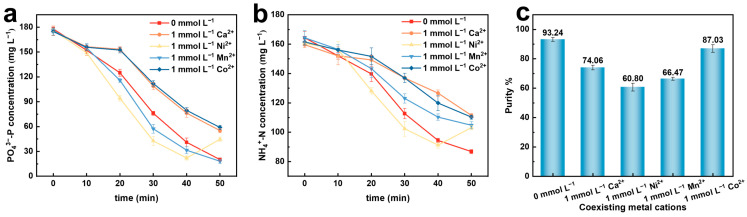
Effects of different coexisting metal cations on the electrochemical recovery of nitrogen and phosphorus. (**a**) PO_4_^3−^–P conversion rate; (**b**) NH_4_^+^–N conversion rate; (**c**) purity of struvite precipitation.

In the process of treating phosphorus-containing wastewater and recovering ammonia nitrogen to form struvite, the presence of coexisting metal cations in actual wastewater should be considered. The presence of metal ions may affect the interactions between ions, and the extent of their interaction with struvite depends on the contents of elements at competitive sites [28]. Therefore, the effects of common coexisting metal cations (including Ca^2+^, Ni^2+^, Mn^2+^, and Co^2+^) [29,30] in phosphate wastewater were studied on the electrochemical precipitation method for preparing struvite. The experimental conditions were as follows: initial current density of 5 mA·cm^−2^; initial pH: 7.0; initial PO_4_^3−^–P concentration: 176 mg·L^−1^. The experimental results are shown in Figure 9.

As shown in Figure 9a, the addition of Ca^2+^ and Co^2+^ resulted in a slight decrease in the recovery rate of phosphate, while the addition of Ni^2+^ and Mn^2+^ led to a certain degree of improvement in the phosphate recovery rate. Additionally, after the addition of coexisting metal cations, the recovery rate of ammonia nitrogen showed varying degrees of decrease, as shown in Figure 9b. When Ca^2+^ is present in the solution, the proportion of Mg^2+^ decreases, possibly leading to competition with Mg^2+^ and competitive reactions with phosphate to form calcium phosphate compounds such as Ca_3_(PO_4_)_2_ precipitates, affecting the purity of struvite [31,32,33]. Ni^2+^ in the solution competes with Mg^2+^, reacts with NH_4_^+^–N and PO_4_^3−^–P, and also undergoes enrichment and adsorption on the surface of struvite, resulting in the precipitation of NiNH_4_PO_4_·6H_2_O (Ni-MAP) and Ni_3_(PO_4_)_2_ [34]. After the addition of Ni^2+^, the concentrations of phosphate and ammonia nitrogen showed increasing trends at the 50th minute of the reaction, which may be due to the easier combination of Ni^2+^ with phosphate to form precipitates, leading to the decomposition of the formed struvite precipitate and thus affecting the purity of the final struvite precipitate. When preparing struvite by electrochemical precipitation in a solution containing Mn^2+^, struvite can act as an adsorbent and adsorb Mn^2+^ into the lattice, inhibiting the growth of struvite by adsorbing Mn^2+^ on the surface of struvite crystals or nuclei, thereby affecting the purity of the struvite precipitate [29]. The addition of Co^2+^ only affected the recovery rate and efficiency of phosphate and ammonia nitrogen and had little effect on the purity of the struvite precipitate [35], as shown in Figure 9c.

**Figure 10:**
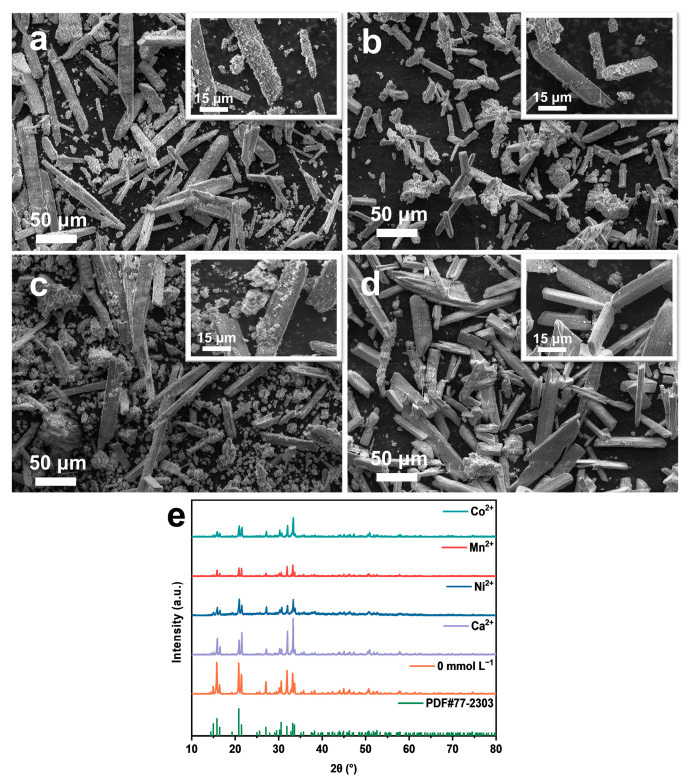
SEM patterns in the presence of different coexisting metal cations: (**a**) Ca^2+^; (**b**) Ni^2+^; (**c**) Mn^2+^; (**d**) Co^2+^. (**e**) XRD patterns of precipitated products.

Figure 10a–d show SEM images illustrating the effects of coexisting metal cations on the precipitate. Figure 10a displays the elongated prismatic crystal structures formed in the presence of Ca^2+^, with many small particles appearing on the surface, which may be precipitates such as Ca_3_(PO_4_)_2_ generated by side reactions [36]. Figure 10b shows the SEM image in the presence of Ni^2+^, which not only affects the structure of struvite crystals but also produces a small number of fine particles on the surface, collectively impacting the purity of struvite due to the co-precipitation of these by-products [37]. In the presence of Mn^2+^, additional blocky particles are generated due to other side reactions between Mn^2+^ and PO_4_^3−^ and OH^−^ in the solution, occurring simultaneously with the crystallization precipitation reaction and competing with each other (Figure 10c) [38]. Figure 10d depicts the SEM image in the presence of Co^2+^, where the precipitate also exhibits elongated prismatic structures similar to those without added metal ions, indicating that the added ions have minimal effect on the crystal structure of struvite, corresponding to the purity of struvite obtained from the tests [39]. Figure 10e shows XRD patterns of the precipitates, revealing a decrease in the peak intensity of each precipitate after the addition of metal ions.

The presence of metal cations can have a greater impact on the purity and morphology of struvite products compared to the absence of added metal cations.

Using the electrochemical precipitation method to prepare struvite for recovering the ammonia-rich solution after NO_3_^−^–RR achieved a good recovery effect and transformed it into valuable struvite resources. At different current densities, nitrogen and phosphorus resources can be effectively recovered, and the purity of struvite is relatively high, indicating the feasibility of this method. Compared to other initial pH values (6.5, 7.5, 8.0, 8.5), a neutral environment is more conducive to the generation of the struvite precipitate. This is because this pH value can achieve the optimal pH value for struvite crystallization and precipitation. In the presence of different initial concentrations of nitrates and nitrites, the purity of the struvite precipitate is hardly affected. This also demonstrates that the method can effectively recover ammonia resources from the rich ammonia solution after nitrate reduction. The presence of coexisting metal ions has little effect on the crystal form of the struvite precipitate, but the purity decreases significantly. This is because metal ions compete with Mg^2+^ in the solution, leading to other side reactions and precipitation. This study demonstrated the feasibility of resource utilization in treating ammonia-containing solutions after the electrochemical reduction of nitrates, reducing phosphate pollution in wastewater, and synthesizing valuable struvite resources, thus achieving economic benefits.

## 3. Potential Implications

The research above shows that the electrochemical precipitation method can efficiently recover and utilize ammonia nitrogen resources after the electrochemical reduction of nitrate and can also effectively treat phosphorus-containing wastewater. Coupling this technology system with the electrochemical nitrate reduction system can improve the economics of the process.

Currently, the international market price of struvite is approximately USD 215.22 per ton of MAP (with a nitrogen content of 5.7%), equivalent to about USD 3.74/kg N, while the price of ammonium nitrogen is only USD 138.60 per ton (with a nitrogen content of 21%), equivalent to about USD 0.69/kg N [40]. Therefore, recycling ammonia nitrogen resources in the form of struvite is economically valuable and environmentally friendly. This technology not only achieves high-value transformation but also produces struvite, which can serve as a source of slow-release, high-quality fertilizer for agricultural applications.

## 4. Materials and Methods

### 4.1. Materials

Ammonium chloride (NH_4_Cl, AR), Dipotassium hydrogen phosphate (K_2_HPO_4_·3H_2_O, AR), Potassium hydroxide (KOH, AR), Hydrochloric acid (HCl, 1 M), Potassium nitrate (KNO_3_, AR), Potassium nitrite (KNO_2_, AR), Anhydrous calcium chloride (CaCl_2_, AR), Nickel chloride (NiCl_2_, AR), Manganese chloride (MnCl_2_, AR), and Cobalt chloride (CoCl_2_, AR) were obtained from Tianjin Kermel Chemical Reagent Co., Ltd. (Tianjin, China).

### 4.2. Characterization

X-ray diffraction (XRD, Rigaku SmartLab SE, Akishima, Japan) with a Cu-Kα radiation source (*λ* = 1.5418 Å) was used to analyze crystals loaded on copper foam. The morphology and chemical compositions of the samples were analyzed using scanning electron microscopy (SEM, TESCAN MIRA LMS, Brno, Czech Republic).

### 4.3. Experimental Apparatus

**Figure 11:**
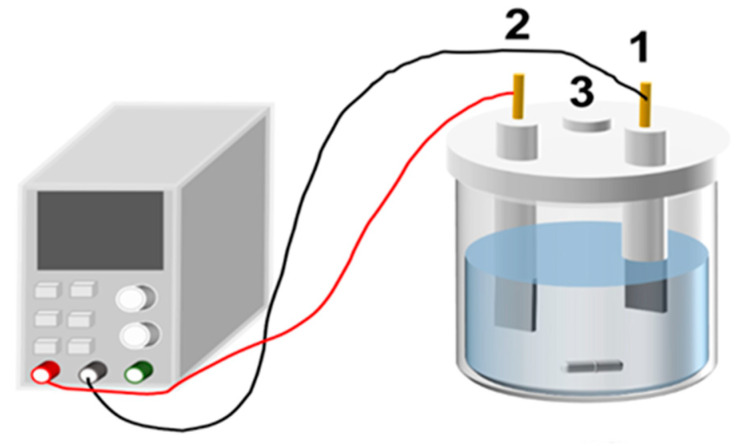
Schematic diagram of experimental apparatus. (1—Cathode; 2—anode; 3—sampling port).

The electrochemical experimental setup used is depicted in Figure 11. The setup mainly consists of a DC power supply, electrolysis cell, thermostatic water bath, and pH meter. The anode employed is a magnesium electrode (composed of magnesium alloy plate with magnesium content of 98%) with dimensions of 20 mm × 20 mm × 2 mm. The cathode is made of stainless steel and has dimensions of 20 mm × 20 mm × 2 mm. The volume of the apparatus is 100 mL. All experiments were conducted using a constant-temperature water bath to maintain a constant temperature of 25 °C.

### 4.4. Experimental Method

For the experiments on electrochemical precipitation for struvite formation, a constant current was provided by a DC power supply. The anode employed is a magnesium electrode, and the cathode is made of stainless steel. The simulated wastewater comprised a mixed solution containing 160 mg N L^−1^ NH_4_Cl and 176 mg P L^−1^ K_2_HPO_4_ in a final volume of 50 mL (The initial ammonia nitrogen concentration was fixed at 160 mg N L^−1^ NH_4_^+^). The experiments were conducted for 50 min at a constant temperature of 25 °C and a stirring rate of 200 rpm. Batch experiments were conducted to investigate the effects of the current density, initial pH, coexisting nitrates and nitrites, and coexisting metal ions on the recovery of nitrogen and phosphorus resources through electrochemical precipitation for struvite formation, and the optimal operating parameters were obtained. Every 10 min, 0.2 mL was taken from the solution for NH_4_^+^–N and PO_4_^3−^–P concentration analysis. The determination of ammonia nitrogen was carried out using the indophenol blue spectrophotometric method, while phosphate analysis was performed using the ammonium molybdate spectrophotometric method. The conversion rates were calculated using the following equations:

(1) $${R(\mathrm{PO}}_{4}^{3-}–P)=\frac{C_{0}{(\mathrm{PO}}_{4}^{3-}–P)-C_{t}{(\mathrm{PO}}_{4}^{3-}–P)}{C_{0}{(\mathrm{PO}}_{4}^{3-}–P)}\;\times \;100\%$$

(2) $${R(\mathrm{NH}}_{4}^{+}–N)=\frac{C_{0}{(\mathrm{NH}}_{4}^{+}–N)-C_{t}{(\mathrm{NH}}_{4}^{+}–N)}{C_{0}{(\mathrm{NH}}_{4}^{+}–N)}\;\times \;100\%$$

where *C*_0_(PO_4_^3−^–P) and *C_t_*(PO_4_^3−^–P) represent the initial concentration and the concentration of PO_4_^3−^–P in the solution (expressed in terms of P) at the time of observation, mg L^−1^. *C*_0_(NH_4_^+^–N) and *C_t_*(NH_4_^+^–N) represent the initial concentration and the concentration of NH_4_^+^–N in the solution (expressed in terms of N) at the time of observation, mg L^−1^.

In the experiment for determining the purity of struvite, the precipitate obtained after the completion of the experiment was filtered and collected. The collected precipitate was dried at room temperature for 48 h. A certain mass of the precipitate was weighed and dissolved in a hydrochloric acid solution. The solution was then diluted to a certain volume and mixed evenly, and the concentration of NH_4_^+^–N in the solution was detected at this point, calculated using Equation (3):

(3) $$\omega =\frac{{C\;\times \;V\;\times \;M}_{\mathrm{MAP}}}{{m\;\times \;M}_{N}}\;\times \;100\%$$

where *C* is the concentration of ammonia nitrogen, mg L^−1^; *V* is the final volume, 50 mL; *M_MAP_* is the molar mass of struvite, 245 g mol^−1^; *m* is the mass of the collected precipitate, mg; and *M_N_* is the molar mass of nitrogen, 14 g mol^−1^.

## 5. Conclusions

In this study, the electrochemical precipitation method was used to efficiently recover ammonia nitrogen and phosphate resources in the form of struvite while achieving the efficient recovery and utilization of the ammonia-rich solution after NO_3_^−^–RR. Under the optimal experimental conditions of a current density of 5 mA·cm^−2^ and an initial pH of 7.0, the phosphate conversion rate was 88.66%, the ammonia nitrogen conversion rate was 47.15%, and the purity of the precipitated struvite reached 93.24%. Additionally, the coexistence of NO_3_^−^–N and NO_2_^−^–N ions in the wastewater after the electrochemical reduction of nitrates has no significant effect on the electrochemical precipitation of struvite for nitrogen and phosphorus recovery, demonstrating the feasibility of this recovery process. However, the presence of metal cations significantly affects the purity of struvite precipitation. This study demonstrates the feasibility of reducing phosphate pollution in wastewater by treating ammonia-containing solutions after nitrate electrochemical reduction, synthesizing valuable struvite resources, and thus obtaining economic benefits, providing a new pathway for the recovery and utilization of ammonia nitrogen resources in NO_3_^−^–RR.

## Data Availability

Data are contained within the article.
